# Reinforcing Small Wins and Frustrating Near-Misses: Further Investigation Into Scratch Card Gambling

**DOI:** 10.1007/s10899-016-9611-0

**Published:** 2016-04-18

**Authors:** Madison Stange, Mikyla Grau, Sandra Osazuwa, Candice Graydon, Mike J. Dixon

**Affiliations:** Department of Psychology, University of Waterloo, Waterloo, ON N2L 3G1 Canada

**Keywords:** Gambling, Scratch cards, Near-misses, Psychophysiology, Arousal, Frustration

## Abstract

Scratch card games are incredibly popular in the Canadian marketplace. However, only recently have researchers started to systematically analyze their structural characteristics and how these in turn affect the gambler. We present two studies designed to further understand the underlying physiological and psychological effects that scratch cards have on gamblers. We had gamblers (63 in Experiment 1, 68 in Experiment 2) play custom made scratch cards involving a small win, a regular loss and a near-miss—where they uncovered two out of the three symbols needed to win the top prize. Our predictions were that despite near-misses and losses being objectively equivalent (the gambler wins nothing) gamblers’ reactions to these outcomes would differ dramatically. During game play, skin conductance levels and heart rate were recorded, as well as how long gamblers paused between each game. Gamblers’ subjective reactions to the different outcomes were then assessed. In both studies, near-misses triggered higher levels of physiological arousal (skin conductance levels and heart rates) than losses. Gamblers paused significantly longer following small wins than other outcomes, and reported high arousal, positive affect and urge to gamble—a constellation of results consistent with their rewarding properties. Importantly near-miss outcomes were rated as highly arousing, negative in emotional tone, and the most frustrating of all three outcome types examined. In Experiment 2, when we measured subjective urge to gamble immediately after each outcome, urge to gamble was significantly higher following near-misses than regular losses. Thus, despite not rewarding the gambler with any monetary gain, these outcomes nevertheless triggered higher arousal and larger urges to gamble than regular losses, a finding that may explain in part, the allure of scratch cards as a gambling activity.

## Introduction

Lottery products are an exceptionally popular form of gambling. For example, in our home jurisdiction of Ontario, Canada, in a single fiscal year (2013/2014) the Ontario Lottery and Gaming Corporation (OLG) reported that lottery sales accounted for approximately 3.3 billion dollars in revenue (OLG 2015). A number of distinct game types exist: lotto (e.g. a traditional lottery where gamblers must wait for a specified amount of time for draws or game outcomes in order to know whether they won or lost), sports games, and INSTANT ticket products, in which prizes are contained on the purchased card itself. INSTANT lottery games are commonly referred to as scratch cards, and involve gamblers uncovering various symbols, numbers, or letters, in the hopes of uncovering a prize (Reid 1986). Of the 3.3 billion dollars alluded to above, over 1 billion was derived from scratch card sales. From 2012/2013 to 2013/2014, scratch card sales increased by 87.2 million dollars (OLG 2015). Clearly, lottery products as a whole are a very popular form of gambling, but scratch cards appear to be trending upward in popularity.

Lottery products are available at almost 10,000 retailers across the province (OLG 2015). These retailers are located in a variety of locations, such as supermarkets, big box retailers, gas stations, and convenience stores. Unlike traditional gambling venues, most of these retailers are regular everyday shopping locations (Papoff and Norris 2009). The demand for these products and their ubiquity in the marketplace make scratch cards an omnipresent gambling medium in Canadian society. However, despite their popularity and availability, surprisingly little is known about how these particular gambling activities impact and influence the gambler.

Of the existing gambling literature examining scratch cards, the majority has looked at the use of these products by youth populations (despite legal restrictions). Indeed, this gambling activity (and lottery products in general) appear to be popular among this demographic (Felsher et al. 2004; Boldero et al. 2010), with many studies reporting high prevalence rates (Donati et al. 2013; Griffiths 2000; Wood and Griffiths 1998). This form of gambling is also known to be popular among adults (Papoff and Norris 2009; Short et al. 2015; Williams et al. 2006). Thus scratch cards are both appealing and popular. Despite some speculation about the potentially harmful effects of these games and their appealing qualities, very few experimental investigations have been undertaken to explore these possibilities further. Thus we pose the following question: what factors and features of these games account for their popularity and widespread use? Additionally, how do these features affect the player?

We propose that the popularity of these games is in large part derived from specific structural features. Our focus on these aspects was guided by research on slot machines—a type of gambling that surprisingly bears many similarities to scratch card gambling (Ariyabuddhiphongs 2011; Griffiths 1995b). A key game feature in slot machine play involves the presence of small, unpredictable wins. The rewarding properties of such wins have been documented using the post-reinforcement pause (PRP). Unlike losing spins, where gamblers tend to spin again immediately, following a win, gamblers pause before triggering the next spin, as though to internally celebrate the win. The length of such pauses varies directly with win size, and has been used to infer the different rewarding properties of these outcomes (Dixon et al. 2013). In a recent study (Stange et al. 2016) we custom-made scratch card games and video recorded gamblers as they played them. Analysis of the videos revealed that players paused longer between games after uncovering a winning outcome than after uncovering losing outcomes. Thus as in slots play, these small wins appear to be rewarding to gamblers and may be one of the specific game features accounting for the popularity of scratch cards.

Slot machines and scratch card games also share a distinctive game feature called a near-miss, an outcome in which a gambler falls just short of a big win (Reid 1986). Consider a three-reel slot machine where three red 7’s on the payline would result in a jackpot win: a near-miss in this game would consist of having two red 7’s land on the payline, with the third just off the payline. Although these outcomes are no different from a regular loss in terms of costing the gambler their wager, near-miss outcomes in slot machines have been shown to prolong slots play (Côté et al. 2003). Additionally, near-miss outcomes have been shown to influence gambler arousal due to frustration. For example, researchers investigating slots gamblers have found that near-misses elicit strong skin conductance responses (SCRs)—even stronger than those for small wins, indicating that a high amount of physiological arousal is associated with these outcomes (Dixon et al. 2011). Additionally, an fMRI investigation of near-misses demonstrated that the mesolimbic reward system was activated by these outcomes, and that near-misses also increased participants’ desire to continue playing the game (Clark et al. 2009). Clearly, despite their objective value as a loss, near-miss outcomes have strong effects on gamblers.

The link between near-misses and gambling urge (i.e. how great an individual’s desire to continue gambling is at a specific point in time) is especially important. Clark et al. (2009) periodically interrupted play in a slots-like game after various outcomes (wins, losses, and near-misses) and polled gamblers about their urge to gamble. They showed that urge was higher following near-misses than regular losses. This finding might not only account for the popularity of slots games, but also might be a feature that could lead to gambling problems.

In a scratch card game, a near-miss outcome would consist of uncovering two of the three jackpot symbols needed to win a large prize. Although researchers have identified near-miss outcomes in scratch cards as a potentially problematic characteristic of this gambling medium (Wood and Griffiths 1998; Reid 1986; Griffiths 1995a, b), only recently have researchers provided actual data showing that scratch card near-misses mimic slot machine near-misses in terms of the physiological and psychological effects they have on gamblers (Stange et al. 2016). In our initial investigation, scratch card near-misses led to the greatest amount of change in gamblers’ skin conductance levels (SCLs) as the symbols of the game were being uncovered. Additionally, near miss outcomes were rated as the most frustrating of the three measured outcomes (small win, loss, and near-miss), and were found to be as subjectively arousing as winning outcomes.

The studies presented here sought to further understand how small wins and near-misses in scratch cards affect gamblers. We sought to replicate our findings that small wins were rewarding outcomes by measuring PRPs, and to provide converging evidence for the arousal-inducing properties of near-misses by supplementing our previous measurements of changes in SCLs with measures of heart rate (HR). We also sought to provide new evidence that both small wins and near-misses promote increases in the urge to continue gambling on scratch card games—a finding that could partially account for their overwhelming popularity.

Based on our previous experiment, the general aim of Experiment 1 was to test the following hypotheses: we predicted that gamblers would show larger PRPs following wins (i.e. pause longer between games to internally celebrate) than following losses or near-misses. For near-misses we predicted that SCLs would increase as gamblers uncovered two of the three jackpot symbols and that such SCL changes would be greater than comparable changes for regular losses (or perhaps even wins). We predicted that we would show similar effects on gambler’s HR, such that HR would be elevated as gamblers successively uncovered the two jackpot symbols. We predicted that near-misses would be more subjectively arousing than regular losses and as arousing as wins, but for different reasons (near-misses should be arousing due to frustration, wins due to the arousal associated with reward). Consequently we predicted frustration ratings should be highest for near-miss outcomes, followed by regular losses, with wins being the least frustrating. Lastly, we predicted that small wins should trigger the urge to continue gambling but so too would near misses (more so than regular losses).

The aim of Experiment 2 was also to confirm the above hypotheses, offering a built-in replication of our findings. As in our previous experiment (Stange et al. 2016), both of the current studies utilized custom made scratch cards presented in a similar format to what consumers would see at an Ontario lottery retailer. Players were shown a display of approximately 100 cards and told that one contained the top prize. They were then instructed to choose two cards from this display that they wished to play during the experiment, in order to closely approximate a realistic gambling experience.

## Experiment 1

### Methods

#### Participants

Sixty-three University of Waterloo undergraduate students were recruited for this experiment and received one course credit in appreciation of their time. The average age of the sample was 20.57 years, with ages ranging from 18 to 36 years (48 females). Participants were all prescreened to ensure that they were: (1) at least 18 years of age (the legal age to purchase lottery products in Ontario), (2) not currently in or seeking treatment for problem gambling, (3) had experience playing scratch cards, (4) not currently in treatment for an anxiety disorder or taking medication for an anxiety disorder (since some anxiolytic medications can interfere with skin conductance recordings), and (5) not allergic or sensitive to gels, adhesives, or sanitizing agents (as such compounds are used in attaching electrodes). All prescreening criteria were confirmed at the time of consent, before the experiment began. One participant who by chance selected the top-prize winning card was excluded from all analyses, and another was excluded from the SCL analyses due to a technical error involving the SCL data.

### Instruments

#### Problem Gambling Severity Index (PGSI)

Following the informed consent procedure, all participants completed the Problem Gambling Severity Index (PGSI), part of the Canadian Problem Gambling Index (Ferris and Wynne 2001). This scale assesses gambling behaviours and attitudes and results in a numerical score from 0 to 27, with scores of 0 indicative of non-problem gambling, 1–4 as low-risk gambling, 5–7 as moderate/at-risk gambling, and scores 8 and above as problem gambling.

#### Subjective Measures of Arousal, Valence, Frustration, and Urge

For each type of scratch card outcome (loss, win, and near-miss), subjective arousal and valence were measured using Self-Assessment Manikins (SAM; Bradley and Lang 1994). Five-point Likert scales were used to assess frustration and urge to gamble. Scores of 1 indicated no frustration or urge to gamble and scores of 5 indicated extreme frustration or urge to gamble.

### Materials

#### Scratch Cards

To closely approximate the scratch card playing experience, participants played custom-made scratch cards designed to mimic a popular scratch card in Ontario, Cash For Life©. The same card design was used for both of the studies presented here to ensure consistency and the comparison of results. The design for these cards was also identical to the design used in our previous investigation of scratch cards and near-misses (Stange et al. 2016). These cards contained scratch off play areas identical to those of real scratch cards.

### Apparatus

#### Display Case

To ensure a realistic playing experience, participants were presented with an array of scratch cards contained within a display case, similar to those found in Ontario lottery retailers. Participants were presented with two pullout trays filled with cards and instructed to choose one card from each tray. In total, the display case held 96 cards, with 1 of these 96 cards containing the top-prize of “Cash for a Month” ($25.00 CAD a week for 4 weeks, totaling $100.00 CAD).

#### Video Recording

Participants’ game play was recorded to allow accurate time locking of physiological responses with outcome delivery. Videos were recorded using the built-in FaceTime camera on a MacBook computer used to record the physiological data. The computer was arranged in a laptop stand such that only participants’ hands and the cards they were scratching were included in the video.

#### SCL Recording

SCL was recorded using non-gelled passive electrodes attached to participants’ index and ring fingers of their non-dominant hand. These electrodes were connected to an ADinstruments PowerLab (model 8/30) with a Galvanic Skin Response amplifier. A 1000 Hz sampling rate was used. LabChart 7.0 analysis software was used to analyze SCLs based on the precise timing of outcome delivery. Specifically, SCL was measured from the time the first symbol of the scratch card game was scratched, to the end of the last symbol in the game being uncovered. This time period was measured for all outcomes that participants experienced.

#### HR Recording

HR was recorded using three electrodes placed on participants skin in a modified Mason-Likar arrangement (Mason and Likar 1966). This arrangement places two electrodes in the infraclavicular fossae 2 cm medial to the deltoid border and a third electrode, acting as an earth ground, on the left anterior abdomen in the anterior axillary line 3–4 cm inferior to the costal margin. These electrodes were also connected to the PowerLab, and a sampling rate of 1000 Hz was used for data collection. HR was analyzed using LabChart software over the same time course as SCL.

### Design

This experiment was a within subjects design. Participants played two scratch cards, with each card containing three separate games. One card contained a loss, a small win of $5.00 CAD, and another loss; the second card contained a loss, the near-miss outcome, and another loss. Thus all participants experienced four losses, one small win, and one near-miss. The order in which the cards were presented (loss/small win/loss card or loss/near-miss/loss card) was counterbalanced.

To closely approximate real scratch card gambling, the rules of our game were similar to those found in existing scratch cards. To win a prize, the participant needed to uncover three matching symbols within one game. Therefore, a win would consist of three matching symbols and three non-matching symbols within the six-symbol matrix. A near-miss outcome would consist of two matching (top-prize) symbols and four non-matching symbols, and a loss would contain six non-matching symbols within the matrix. The symbols in our game, like those in Cash for Life© were monetary amounts, with the exception of the top prize amount, which was denoted by the word “MONTH”. This was chosen to emulate the “LIFE” symbol in Cash for Life©. Thus, the near-miss outcome contained only two of the three “MONTH” symbols needed to win the top prize, and the small win consisted of three matching $5.00 symbols, interspersed among other non-matching symbols.

### Procedure

At the beginning of the Experiment, all participants read and signed an informed consent letter. The University of Waterloo’s Office of Research Ethics approved all procedures in this Experiment. Following consent, participants completed the PGSI on a laptop computer using Qualtrics survey software. Next the participant selected the scratch cards that they would be playing in the game play portion of the Experiment. Participants were informed that they would be choosing two cards, and that one of the cards within the display case contained the top prize of Cash for a Month, equivalent to $100.00 CAD. The researcher removed both pullout card trays, presented them to the participant, and allowed them to freely choose one card from each tray.

Once they had selected their cards, the researcher explained the rules of the game. For clarification purposes, the researcher used an oversized version of the scratch card game as an example. The researcher explained that each scratch card contained three separate games, and that each game contained six symbols. Participants were told that to win a prize, they had to uncover three matching symbols within one game. The matching symbols denoted the prize that was won. The researcher also explained that to win the top prize of Cash for a Month, three “MONTH” symbols had to be uncovered within a given game. Participants were also asked to scratch each game in three rows, starting from the top, and moving from the leftmost symbol to the rightmost symbol (to ensure consistent outcome delivery between participants). Additionally, they were asked to only move onto the next game once they were finished scratching the preceding game in its entirety.

After choosing their cards and going over the rules of the game, participants were escorted to another lab room to wash their hands with Ivory soap to ensure clear SCL recordings. Participants were shown how to attach the three HR electrodes and given a mirror, a reference diagram, and a private space behind a curtain to apply them. A same-sex experimenter was available if participants needed assistance with electrode application. Next, the SCL electrodes were attached to the upper phalanges of the first and third fingers of the participants’ non-dominant hand. Participants were instructed to keep their non-dominant hand as still as possible while they were scratching the cards in an attempt to limit the amount of movement artifacts in the HR and SCL data.

Following this set-up phase, the researcher placed one of the cards that the participant had chosen into a secure scratching platform (see Stange et al. 2016). This ensured that the participant could scratch the card with only one hand and also provided a complete video recording of the card surface. The scratching platform was angled at approximately 30° to ensure participant comfort during game play. Participants were provided with a small metal washer (2.2 cm in diameter) with which to scratch the cards.

Players completed game play of the first card. The second card was inserted into the scratching platform, and players completed game play of the second card. They then were shown exemplars of the different types of outcomes (a loss, a win, and a near-miss) and answered the subjective arousal, valence, frustration and urge questions to gauge their subjective reactions to these displayed outcomes.

### Data Reduction

Recall that the game orders were as follows: loss, small win, loss, and loss, near-miss, loss, with the order of card presentations counterbalanced. For all analyses, the comparisons of interest were reactions to the win, reactions to the near-miss, and reactions to a loss. Although there were four losing outcomes, only one of these was selected for analysis, namely the first loss on the second card played by the participant. By choosing this epoch we ensured that for each analyzed outcome the proceeding epoch was a loss. As such any effects of outcome type shown in the analysis could not be differentially affected by the previous outcome (since they were always losses). Thus for all analyses with all participants there were three data points (one win, one near-miss and one loss).

For the SCL data, the recording epoch started from the time they began scratching the first symbol in the game matrix to the time they uncovered the last symbol revealing the outcome. To assess whether there were changes in SCL levels as gamblers revealed the symbols (e.g. did SCLs go up as gamblers sequentially uncovered the “MONTH” symbols in the near-miss game), we used LabChart software to record the slope of the SCLs over the entire recording epoch. For each participant the slope of the winning outcome, the near-miss outcome, and the first loss on card 2 was calculated.

For HR, we used the same epochs as above, and calculated the beats per minute in the winning, near-miss and loss epochs. For PRPs, the time between outcome delivery (uncovering the last symbol in a particular game) and the initiation of the next game (scratching the first symbol in the next game) was recorded.

### Analytical Strategy

The data was subjected to an outlier trimming procedure using a cutoff of three standard deviations from the mean of that outcome condition. The remaining data were then submitted to a repeated-measures analyses of variance (ANOVA), with outcome type as the repeated measures factor. Follow up analyses were conducted using paired-samples *t* tests (Fischer’s least significant difference test [LSD]). For all repeated measures analyses, in the event of sphericity assumptions being violated (assessed using Mauchly’s test), a Greenhouse-Geisser correction was applied prior to calculating the F-ratios. In these instances, corrected degrees of freedom are reported.

### Results

#### PGSI

Participants’ scores on the PGSI classified 41 participants as non-problem gamblers, 20 participants as low-risk, 1 participant as a moderate/at-risk gambler, and no problem gamblers. Note that the PGSI was used to characterize our sample—no specific predictions were made concerning gambling status.

#### Pre-outcome SCLs

Data from one participant could not be analyzed due to a recording error. Table 1 shows that for the remaining participants, losing outcomes were generally associated with negative slopes, but both winning and near-miss outcomes showed increases in SCLs over time (i.e. positive slopes). Statistically, there was a main effect of outcome *F*(2, 118) = 18.45, *p* < .001, η^2^ = .238. Post hoc Fisher’s LSD comparisons indicated that winning slopes were steeper than losing slopes *t*(59) = 4.31, *p* < .001, near-miss slopes were steeper than losing slopes, *t*(59) = 5.59, *p* < .001, but that there was no difference between winning and near-miss slopes *t*(59) = −1.57, *p* = .12.

**Table 1:** Mean psychophysical (SCL, HR), behavioural (PRP) and subjective (arousal, valence, frustration, and urge) values for the three game outcomes in Experiment 1 (standard deviations in parentheses)

Dependent variables	Outcome type		
	Win	Near-miss	Loss
SCL slopes	0.12^−5^ (0.34^−4^)	0.11^−4^ (0.34^−4^)	−0.27^−4^ (0.36^−4^)
HR	88.74 (12.25)	89.82 (12.04)	86.0 (12.22)
PRP	3.80 (2.70)	2.91 (1.92)	2.34 (1.81)
Arousal	3.15 (0.97)	2.74 (0.89)	2.03 (0.72)
Valence	4.31 (0.74)	2.82 (0.78)	2.92 (0.64)
Frustration	1.17 (0.38)	1.90 (0.73)	1.57 (0.56)
Urge	2.16 (0.82)	2.0 (0.82)	1.97 (0.88)

*SCL* skin conductance level, *HR* heart rate, *PRP* post-reinforcement pauses

#### Heart Rate

Data from 24 participants could not be analyzed due to excessive movement artifacts associated with the scratching movements during play. For the remaining 38 participants, the average BPM was calculated for winning, near-miss and losing epochs. Table 1 shows elevated heart rates for wins and near-misses compared to losses. A repeated measures ANOVA revealed a main effect of outcome *F*(2, 74) = 20.36, *p* < .001, η^2^ = .355. Fischer’s LSD t-tests demonstrated that HR was significantly higher across winning epochs than losing epochs *t*(37) = 4.75, *p* < .001. Near-miss epochs had higher HR than losing epochs *t*(37) = 6.59, *p* < .001, but there were no significant differences in HR for winning versus near-miss epochs. *t*(37) = −1.57, *p* = .13.

#### Post-reinforcement Pauses

Table 1 shows the highest PRPs for wins, followed by near-misses, with the shortest PRPs following regular losses. A repeated measures ANOVA on PRPs revealed a main effect of outcome type, *F*(2, 118) = 15.58, *p* < .001, η^2^ = .209. The PRP was significantly longer following wins versus losses, *t*(59) = 5.41, *p* < .001, and wins versus near-misses, *t*(59) = 3.13, *p* = .003. These t-tests also showed that PRP’s following near-misses were marginally longer than for losses, *t*(59) = 2.43, *p* = .018.

#### Subjective Measures: Arousal, Valence, Frustration, and Urge

Subjective arousal ratings in Table 1 show the highest arousal for wins, and lowest arousal for losses, with near-misses falling in between. Arousal ratings (assessed with SAMs) revealed a significant main effect of outcome, *F*(2, 122) = 59.10, *p* < .001, η^2^ = .492. Paired samples t-tests revealed significantly higher subjective arousal ratings for winning outcomes compared to losing outcomes, *t*(61) = 10.07, *p* < .001, and near-miss outcomes, *t*(61) = 3.70, *p* < .001. Additionally, near-misses were rated as higher in subjective arousal than losses, *t*(61) = 7.87, *p* < .001.

Valence ratings in Table 1 show the most positive ratings for wins, with equivalently low ratings for near-misses and losses. There was a significant main effect of outcome, *F*(1.82, 110.95) = 94.44, *p* < .001, η^2^ = .608 (since the assumption of sphericity was violated Mauchly’s test: *X* ^*2*^(2) = 6.30, *p* = .043, a Greenhouse-Geisser correction was applied). Winning outcomes were rated as significantly more positive in valence than both losing outcomes, *t*(61) = 11.33, *p* < .001, and near-miss outcomes *t*(61) = 10.95, *p* < .001. There were no significant differences between losing and near-miss outcomes, *t*(61) = .948, *p* = .35.

Table 1 shows that the highest frustration ratings were for near-misses, more so than for losses and wins. An ANOVA on frustration ratings revealed a main effect of outcome, *F*(2, 118) = 36.96, *p* < .001, η^2^ = .385. Paired samples t-tests revealed that near-miss outcomes were rated as significantly more frustrating than wins, *t*(59) = 7.75, *p* < .001, and (most importantly) significantly more frustrating than losing outcomes, *t*(59) = 3.94, *p* < .001. Finally, losing outcomes were rated as more frustrating than winning outcomes, *t*(59) = 5.27, *p* < .001.

The final subjective measure, urge to continue gambling, revealed what appear in Table 1 to be smaller effects (compared to the other measures). There was a main effect of outcome, *F*(2, 120) = 3.82, *p* = .025, η^2^ = .06. Paired samples t-tests revealed that winning outcomes were associated with a higher rating of urge to continue gambling compared to losing outcomes, *t*(60) = 2.56, *p* = .013, and compared to near-miss outcomes, *t*(60) = 2.10, *p* = .04. No significant difference was found between the urge ratings for losing and near-miss outcomes, *t*(60) = −.444, *p* = .66.

### Discussion

Both SCL and HR measures showed that revealing the symbols that lead to a regular loss was an experience that was the least physiologically arousing. By contrast, revealing the symbols in both winning and near-miss outcomes triggered significantly greater arousal. Here the contrast between losses and near-misses is paramount, as these outcomes are objectively equivalent in that the player gains nothing, yet they generate very different physiological responses. Consistent with research in slot machine play (Dixon et al. 2011), near-misses triggered more frustration than ordinary losses. However, unlike gamblers’ reactions to slot machine near-misses, scratch card near-misses did not lead to increases in the urge to continue gambling. One possible reason why we failed to show increases in urge following near-misses is a methodological one: since we only measured gamblers’ reactions to outcomes after completing the scratch cards, there were delays between the actual in-game outcome and when we gathered reactions to that outcome. We reminded the participant of the game outcome, and essentially asked them to remember how they felt. Thus remembered urge may have differed or become attenuated from the urge that gamblers felt during game play. In Experiment 2, gamblers played each game, and were immediately polled about their game experiences. By gathering gamblers’ reactions immediately after each outcome we hoped to better capture any increase in the urge to gamble following near-misses.

## Experiment 2

All procedures were identical to those in Experiment 1, with two exceptions: (1) we added subjective measures assessing positive and negative valence, as well as disappointment, and used Likert scales for all subjective questions (see Materials section), and (2) immediately after gamblers completed an outcome, they pressed a button on a response box indicating they were ready to answer questions pertaining to that outcome. Subjective assessments of that outcome were then administered. Thus subjective assessments were gathered immediately after each experienced outcome.

### Methods

#### Participants

Sixty-eight University of Waterloo undergraduate students were recruited for this experiment and received one course credit in appreciation of their time. The average age of the sample was 19.87 years, with age ranging from 18 to 35 (44 females). Participants were prescreened to ensure that they met inclusion criteria (defined in Experiment 1). Of the 68 students recruited, four participants were excluded from all analyses due to idiosyncratic, non-left-to-right scratching patterns, and another three were excluded from SCL and HR analyses due to technical errors. One additional participant was excluded from HR, SCL, and PRP analyses due to a video recording error compromising the analysis of time-locked responses.

### Instruments

#### PGSI

As in Experiment 1, all participants completed the PGSI as part of the Canadian Problem Gambling Index (Ferris and Wynne 2001).

#### Subjective Measures of Arousal, Positive and Negative Valence, Frustration, Disappointment, and Urge

Following each outcome, participants answered 6 questions assessing various subjective aspects of scratch card play. The questions assessed the following dimensions: arousal, positive mood, negative mood, disappointment, frustration, and urge to gamble. The questions were presented in Likert scale format, with response options ranging from 1 to 5 with 1 representing the absence of the feeling or dimension in question, and 5 representing extreme or strong presence of the feeling or dimension. The questions were read aloud once to participants during the first question set and displayed on the wall in front of the participant should they need a reference or clarification on wording at any point in the question sets. Participants gave their answers verbally, with the researcher providing a question prompt (e.g. “arousal?”) for the participant and then recording the participant’s verbal response (i.e. a number between 1 and 5). Four unique question orders were created and randomly assigned to each participant to account for order effects.

### Materials

#### Scratch Cards

The scratch cards used in this experiment were identical to the cards used in Stange et al. (2016) and Experiment 1 of the current paper.

### Apparatus

#### Display Case, Video Recording, SCL Recording, and HR Recording

This experiment utilized the same apparatus as in Experiment 1.

### Procedure

Procedures were identical to Experiment 1 except that immediately after completing each game, participants were instructed to press a button placed next to the scratching area. Once the button press had been completed, subjective questions were answered verbally. Participants responded to all 6 subjective scales (arousal, positive valence, negative valence, frustration, disappointment, and urge to continue gambling) immediately following each outcome. This interleaved pattern of game play, followed by subjective questions continued for all six games.

### Results

#### PGSI

Participants’ scores on the PGSI revealed that 41 participants were non-problem gamblers, 21 participants as low-risk, and 2 participants as moderate/at-risk gamblers. No problem gamblers were identified within this sample.

#### Pre-outcome SCLs

Average slopes of SCLs over the time leading up to outcome delivery are shown in Table 2. Here all slopes were negative, however of the three outcomes, the near-miss outcome appeared to be the least negative. A repeated measures ANOVA revealed a main effect of outcome, *F*(1.81, 106.50) = 4.58, *p* = .015, η^2^ = .072. Paired samples t-tests revealed that SCL’s leading up to losing outcomes were not significantly different from those leading up to wins, *t*(59) = −1.10, *p* = .278. SCL’s leading up to near-miss outcomes were significantly greater than those leading to winning outcomes, *t*(59) = 2.09, *p* = .041, and those leading to losing outcomes, *t*(59) = 2.59, *p* = .012.

**Table 2:** Mean psychophysical (SCL, HR), behavioural (PRP) and subjective (arousal, positive and negative valence, frustration, disappointment, and urge) values for the three game outcomes in Experiment 2 (standard deviations in parentheses)

Dependent variables	Outcome type		
	Win	Near-miss	Loss
SCL slopes	−0.21^−4^ (0.31^−4^)	−0.10^−4^ (0.37^−4^)	−0.27^−4^ (0.42^−4^)
HR	88.59 (10.42)	88.38 (9.38)	86.73 (9.87)
PRP	3.95 (2.23)	3.53 (1.82)	2.85 (1.39)
Arousal	3.47 (0.99)	2.81 (0.96)	2.33 (0.91)
Positive valence	3.72 (1.03)	2.58 (1.01)	2.50 (0.98)
Negative valence	1.22 (0.45)	1.83 (0.92)	1.63 (0.78)
Frustration	1.18 (0.39)	2.02 (1.03)	1.76 (0.88)
Disappointment	1.13 (0.34)	2.58 (1.29)	2.10 (1.08)
Urge	2.22 (1.25)	2.16 (1.20)	1.88 (1.05)

*SCL* skin conductance level, *HR* heart rate, *PRP* post-reinforcement pauses

#### Heart Rate

Data from 18 participants could not be analyzed due to excessive movement artifacts. As shown in Table 2 the data from the remaining 42 participants indicated that uncovering wins and near-misses appeared to be more arousing than uncovering losses. A repeated measures ANOVA revealed a main effect of outcome, *F*(1.70, 69.57) = 5.17, *p* = .01, η^2^ = .112. Subsequent paired samples t-tests showed that the average HR for near-miss outcomes and winning outcomes were not significantly different, *t*(41) = .28, *p* = .78. However, average HR leading up to wins was significantly higher than HR leading up to losses, *t*(41) = 3.38, *p* = .002. Likewise, HR leading up to near-misses was significantly greater than for losses *t*(41) = 2.87, *p* = .006.

#### Post-reinforcement Pauses

The PRPs in Experiment 2 were the pause durations between the outcome reveal to pressing the button to initiate the answering of the subjective questions. Table 2 shows the longest pauses following wins, and the shortest for losses, with near-misses falling in between. A repeated measures ANOVA revealed a main effect of outcome type, *F*(1.71, 99.27) = 10.19, *p* < .001, η^2^ = .149. Wins led to longer PRPs than losses *t*(58) = 4.50, *p* < .001. PRPs were longer following near-misses compared to losses, *t*(58) = 3.44, *p* = .001. Interestingly, there were no significant differences between PRPs following wins and near-misses, *t*(58) = 1.46, *p* = .15.

#### Subjective Measures: Arousal, Positive and Negative Valence, Frustration Disappointment, and Urge

Subjective arousal ratings (as shown in Table 2) were highest for wins, somewhat lower for near-misses, and lowest for losses. The ANOVA revealed a main effect of outcome, *F*(1.77, 111.28) = 49.50, *p* < .001, η^2^ = .440. Wins were significantly higher in subjective arousal than near-miss outcomes, *t*(63) = 5.27, *p* < .001, and losing outcomes, *t*(63) = 9.07, *p* < .001. Subjective arousal ratings of near-miss outcomes were significantly higher than losing outcomes, *t*(63) = 5.28, *p* < .001.

Table 2 shows that positive valence was highest for wins, with near-misses and losses showing little difference. A repeated measures ANOVA revealed a main effect of outcome type *F*(1.80, 113.29) = 64.98, *p* < .001, η^2^ = .508. Wins were higher in positive valence than both losses, *t*(63) = 9.60, *p* < .001, and near-misses, *t*(63) = 8.67, *p* < .001. Near-misses and losses did not differ significantly, *t*(63) = −.80, *p* = .428.

Subjective negative valence ratings were highest for near-misses and lowest for wins. There was a main effect of outcome type, *F*(1.74, 102.45) = 19.40, *p* < .001, η^2^ = .247. Near-misses were rated as significantly more negative than losses *t*(59) = 2.19, *p* = .033, and significantly more negative than wins *t*(59) = 5.18, *p* < .001. Additionally, losing outcomes were rated as significantly more negative than winning outcomes, *t*(59) = 4.64, *p* < .001.

Table 2 shows that near-misses were the most frustrating of all three outcome types. There was a main effect of outcome type, *F*(1.78, 108.71) = 25.32, *p* < .001, η^2^ = .293. Near-miss outcomes were rated as significantly more frustrating than both winning, *t*(61) = 5.98, *p* < .001, and losing outcomes, *t*(61) = 2.34, *p* = .022. Losses were also rated as more frustrating than winning outcomes, *t*(61) = 5.32, *p* < .001.

Subjective ratings of disappointment showed a similar pattern. There was a main effect of outcome type, *F*(1.61, 98.37) = 58.82, *p* < .001, η^2^ = .491. As shown in Table 2, near-miss outcomes were rated as significantly more disappointing than winning outcomes, *t*(61) = 8.78, *p* < .001, and more disappointing than losing outcomes, *t*(61) = 4.41, *p* < .001. Losses were rated as more disappointing than winning outcomes, *t*(61) = 7.56, *p* < .001.

For urge, Table 2 shows that (as in Experiment 1) wins led to the highest urge to gamble, but now ratings of near-misses were also elevated. A repeated measures ANOVA revealed a main effect of outcome, *F*(2, 126) = 7.87, *p* = .001, η^2^ = .111. Wins were rated as provoking a stronger urge to continue gambling than losses, *t*(63) = 3.72, *p* < .001, but not near-misses, *t*(63) = .683, *p* = .497. Crucially near-misses led to higher urges to gamble than losses, *t*(63) = 3.02, *p* = .004.

### Discussion

The majority of our findings from Experiment 1 were replicated in Experiment 2. Specifically, as in Experiment 1, HR was elevated leading up to winning and near-miss outcomes, and lowest leading up to regular losses. SCLs in Experiment 2 showed an overall downward trend throughout outcome delivery; however, the least negative SCLs were found leading up to near-miss outcomes, and these SCLs were significantly less negative than those for regular losses, thus replicating the pattern found in both our previous investigation (Stange et al. 2016) and in Experiment 1. In Experiment 2, participant’s behaviour during game play, as measured by PRPs, showed an interesting pattern of results. Players paused longer following small wins and near-misses, with the shortest pauses taking place following losing outcomes. Participant’s subjective ratings of the outcome types help explicate some of the observed behavioural and physiological results. Small wins were rated as the most subjectively arousing outcomes, the most positive in valence (and the least negative), and the lowest in frustration and disappointment. Near-misses were rated as moderately subjectively arousing, highest in negative valence, and highest in subjective frustration and disappointment. Regular losses were rated as the least subjectively arousing, moderately negatively valenced, and moderately frustrating and disappointing. Interestingly, in Experiment 2, we observed an equal amount of urge to continue gambling generated for both small win and near-miss outcomes. Losses were rated as significantly lower in urge to continue gambling compared to these two outcome types. Thus the modification of when we polled the subjective reactions of players (immediately after the outcomes in Experiment 2) seems to have allowed a clearer and more accurate gauge of the subjective experience of scratch card play.

## General Conclusions

Although scratch cards are a popular and remarkably prevalent form of gambling in today’s marketplace, very little research has addressed how these products affect the gambler. In Experiment 1 SCLs rose as gamblers uncovered the symbols in both the winning and near miss outcomes, whereas for losses the slopes of SCLs over time were negative. This replicates the pattern of SCLs observed in our original investigation (Stange et al. 2016). Here the most important contrast is between near-misses and losses, as even though both outcomes are objectively monetary losses, they appear to be treated very differently in terms of the arousal that they generate. In Experiment 2, we replicated this relationship. Although there seemed to be a general decline in arousal across all epochs, the rate of decline was significantly shallower for the near-misses than for the losses. Although it is unclear why in Experiment 2 SCLs appeared to decline in all conditions, the smallest declines being in the near-miss condition and the greatest declines in the loss condition are consistent with the interpretation that participants experience near-misses differently than losses.

In Experiments 1 and 2, HR provided converging evidence for the arousal-inducing properties of near-misses. In both Experiments HR was higher in the near-miss condition than the loss condition. Interestingly, in both Experiments, HR in the near-miss conditions were as elevated as in the small win condition when gamblers actually won a prize. In line with these results, nearly 40 years ago the UK’s Report of the Royal Commission on Gambling deemed scratch cards “heart stoppers” (Moran 1979), expressing concern that these games “give the illusion of coming close to winning a big prize” (Moran 1979, p. 7). The data presented here confirm the long-suspected impact of near-misses on arousal that other authors have postulated (Reid 1986; Griffiths 1995a, b). Physiologically, near-miss outcomes elevate both SCL and HR. The subjective reports concerning arousal provide even further evidence for this relationship: in both studies near-misses were rated as being significantly more arousing than losses.

The rewarding property of small wins was most clearly evident in our analyses of PRPs. In our previous study (Stange et al. 2016) and in both experiments presented here, small wins led to significantly longer pauses between games than losses. Our interpretation of such pauses is that they are linked to reward: gamblers pause following wins to internally celebrate these outcomes. These same reward related pauses following wins are seen in slots gamblers (Dixon et al. 2013). Consistent with this interpretation, winning outcomes triggered the highest valence ratings (i.e. were rated the most positive) in Experiment 1. This finding was replicated in Experiment 2 where wins were associated with the highest positive valence ratings and lowest negative valence ratings (as well as the lowest frustration and disappointment scores). Collectively this data lends credence to our contention that the long PRPs following wins are related to the rewarding properties of these winning outcomes.

The relationship between near-misses and PRPs was more complex. In Experiment 1, near-misses had smaller PRPs than wins, whereas in Experiment 2, the PRPs of near-misses and wins were actually equivalent. The subjective ratings indicate that it would be erroneous to interpret the elevated PRPs for near-misses as related to the enjoyment aspect of the reward system. In both studies near-misses were rated as the most frustrating outcome; they were also the most disappointing outcome in Experiment 2 (disappointment was not measured in Experiment 1). One interpretation of the near-miss PRP data is that it was during the PRP period following a near-miss that gamblers were ruminating on how frustratingly close they were to the grand prize and how disappointed they felt at not having won. Such ruminations may have elevated near-miss PRP lengths over and above those of regular losses.

Arguably the most important finding in this series of studies involves the urge to gamble. In both studies small wins triggered the urge to gamble—a finding that may account for the popularity of scratch cards. In our studies, any time a gambler won a small reward, they experienced the urge to play again. It is not unreasonable to assume that a substantial number of gamblers may act on this urge, and purchase more scratch cards with their winnings in a real gambling environment.

In slot machine research near-misses have been shown to trigger the urge to continue gambling. Clark et al. (2009) periodically interrupted slots play after losses, wins, and near-misses and assessed gambler’s urge to gamble. They showed that despite being a kind of loss, near-misses prompted greater urge to gamble than regular losses. In Experiment 1 we failed to replicate this finding, but noted that this might have been due to a lengthy delay between when the actual outcome occurred, and when we polled participants about their subjective experiences. In Experiment 2 when we polled participants about their urge to gamble immediately after each of the outcomes (wins, losses, and near-misses), we now showed that near-misses created higher urges to gamble than regular losses. This finding is important since it shows a way of potentially increasing scratch card purchases without any costs to the scratch card providers. Thus near-misses may be a second structural feature that accounts for the popularity of this gambling activity.

## Limitations and Future Directions

While the present studies help shed light on what currently is an under-researched area of gambling behaviour, there are still many questions unanswered. For example why were all SCL slopes negative in Experiment 2 (albeit less negative for near-misses)? Perhaps the procedural differences between the studies are partially responsible: asking participants to gauge their affective responses on a number of dimensions *immediately after* game play may have elevated their physiological arousal—with a reduction in SCLs occurring as they scratched the symbols in the next game. It also remains unclear if near-miss outcomes affect urge to continue gambling above and beyond the other outcome types. While our hypotheses based on slot machine literature predicted that this would be the case, our data suggest otherwise and draw attention to the fact that small wins found in this form of gambling may be more influential than we imagined.

Other limitations include the fact that participants were not investing their own money in the experiment, as would be the case in the real-world. Additionally, the jackpot prize of our game was significantly smaller than those found in real scratch card games. Yet despite this, our results still indicated significant effects of near-miss outcomes on arousal and the urge to continue gambling. Perhaps these effects would be more pronounced if gamblers were using their own money or had the chance of winning an even larger prize. In line with this notion, it is also important to consider that we only measured *urge* to continue gambling—it remains unclear whether or not this urge would translate into the repurchasing of more scratch cards, possibly illustrating the potentially detrimental effects that these types of outcomes may have on real-world gambling behaviour. Additionally, our understanding of these gambling activities would be deepened by analyzing the influence of gambling frequency (in general, or specifically the frequency of scratch card play), problem gambling severity, or participant gender on the physiological and subjective effects of scratch card play, and future research studies designed to target a population with a wider range of gambling problems than a sample of typical University students could answer these questions.
